# The transcription factor *Zfp503* promotes the D1 MSN identity and represses the D2 MSN identity

**DOI:** 10.3389/fcell.2022.948331

**Published:** 2022-08-23

**Authors:** Zicong Shang, Lin Yang, Ziwu Wang, Yu Tian, Yanjing Gao, Zihao Su, Rongliang Guo, Weiwei Li, Guoping Liu, Xiaosu Li, Zhengang Yang, Zhenmeiyu Li, Zhuangzhi Zhang

**Affiliations:** Key Laboratory of Birth Defects, Children’s Hospital of Fudan University, State Key Laboratory of Medical Neurobiology and MOE Frontiers Center for Brain Science, Institutes of Brain Science, Fudan University, Shanghai, China

**Keywords:** ZFP503, striatum, fate switch, LGE, medium-sized spiny neurons

## Abstract

The striatum is primarily composed of two types of medium spiny neurons (MSNs) expressing either D1- or D2-type dopamine receptors. However, the fate determination of these two types of neurons is not fully understood. Here, we found that D1 MSNs undergo fate switching to D2 MSNs in the absence of *Zfp503*. Furthermore, scRNA-seq revealed that the transcription factor *Zfp503* affects the differentiation of these progenitor cells in the lateral ganglionic eminence (LGE). More importantly, we found that the transcription factors *Sp8/9*, which are required for the differentiation of D2 MSNs, are repressed by *Zfp503*. Finally, sustained *Zfp503* expression in LGE progenitor cells promoted the D1 MSN identity and repressed the D2 MSN identity. Overall, our findings indicated that *Zfp503* promotes the D1 MSN identity and represses the D2 MSN identity by regulating *Sp8/9* expression during striatal MSN development.

## Introduction

The striatum, the major component of the basal ganglia, consists of the caudate and putamen in humans. The majority of striatal neurons are medium-sized spiny neurons (MSNs), which can be further divided into two cell types: one cell type that expresses the dopamine receptor DRD1 (D1 MSNs) and another cell type that expresses the dopamine receptor DRD2 (D2 MSNs). D1 MSNs directly project to the substantia nigra pars reticulata to form the direct pathway, whereas D2 MSNs give rise to the indirect pathway, which projects to the external part of the globus pallidus (Gerfen et al., 1990; Kawaguchi et al., 1995; Gerfen and Surmeier, 2011). Although a subset of MSNs co-express DRD1 and DRD2 receptors, the proportion of these neurons is very small, especially in dorsal striatum (Gerfen et al., 1990; Bertran-Gonzalez et al., 2008). Dysfunction of the striatum is closely associated with multiple neuropsychiatric diseases, including Huntington’s disease (HD), Parkinson’s disease (PD), schizophrenia and obsessive-compulsive disorder/attention deficit hyperactivity disorder (DeLong, 1990; Wichmann and DeLong, 1996; Gerfen and Surmeier, 2011). The diversity of striatal function is critically dependent on the normal development of MSNs at the embryonic stage. Previous studies have shown that the lateral ganglionic eminence (LGE), the primordium of the striatum, contains two distinct regions: a dorsal region (dLGE) and a ventral region (vLGE). The dLGE generates most interneurons of the olfactory bulb. In contrast, the vLGE mainly gives rise to striatal MSNs (Olsson et al., 1995; Anderson et al., 1997; Yun et al., 2001; Stenman et al., 2003; Waclaw et al., 2006; Wen et al., 2021). Initially, the transcription factors (TFs) *Gsx1/2*, *Ascl1*, *Dlx1/2* and *Meis2* control the differentiation of progenitors into immature MSNs (Anderson et al., 1997; Yun et al., 2003; Long et al., 2009; Waclaw et al., 2009; Wang et al., 2009; Pei et al., 2011; Wang et al., 2013; Chapman et al., 2018; Su et al., 2022). Although D1 MSNs and D2 MSNs share many properties, they specifically express different molecules to fate acquisition (Lobo et al., 2006; Gokce et al., 2016; Stanley et al., 2019; Puighermanal et al., 2020). Our previous studies showed that the TFs *Sp8/9* and *Six3* are specifically required for the differentiation of most D2 MSNs (Zhang et al., 2016; Xu et al., 2018; Song et al., 2021). Although many studies have reported that some TFs control the differentiation of subpopulation of the D1 MSNs, such as *Zfhx3*, *Ebf1*, *Isl1* and *Foxo1* (Garel et al., 1999; Lobo et al., 2008; Ehrman et al., 2013; Lu et al., 2014; Waclaw et al., 2017; Zhang et al., 2019), the TFs that specifically guide the differentiation of most D1 MSNs still need to be further explored. To date, two studies have indicated that the transcription factor *Zfp503* is required for the differentiation of the most D1 MSNs (Chen et al., 2020; Soleilhavoup et al., 2020). However, the mechanism involved in these processes is still largely unknown.

Here we investigated the role of *Zfp503* in the development of striatal MSNs. *Zfp503* is specifically expressed in most striatal MSNs at later stages of striatal development. We used *Sp9-Cre* and *Dlx2-Cre* to knock out *Zfp503* expression in the ganglionic eminences (GEs) and found switching of D1 MSNs to D2 MSNs in *Zfp503* conditional knockout (*Zfp503*-CKO) mice. Furthermore, our scRNA-seq revealed that D1 MSNs mature faster (at E14.5) than D2 MSNs. At the cell population level, we found that progenitor cells and D2 MSNs were increased but D1 MSNs were significantly decreased in the LGE of *Zfp503*-CKO mice. Finally, we found that the TFs *Sp8/9* were significantly increased in the LGE of *Zfp503*-CKO mice. *Zfp503* gain of function experiments showed promotion of the D1 MSN identity and repression of the D2 MSN identity. Taken together, our findings suggest that the TF *Zfp503* promotes D1 MSN cell identity and represses D2 MSN cell identity by regulating *Sp8/9* expression during striatal development.

## Results

### D1 MSNs are converted into D2 MSNs in *Zfp503*-CKO mice

**FIGURE 1:**
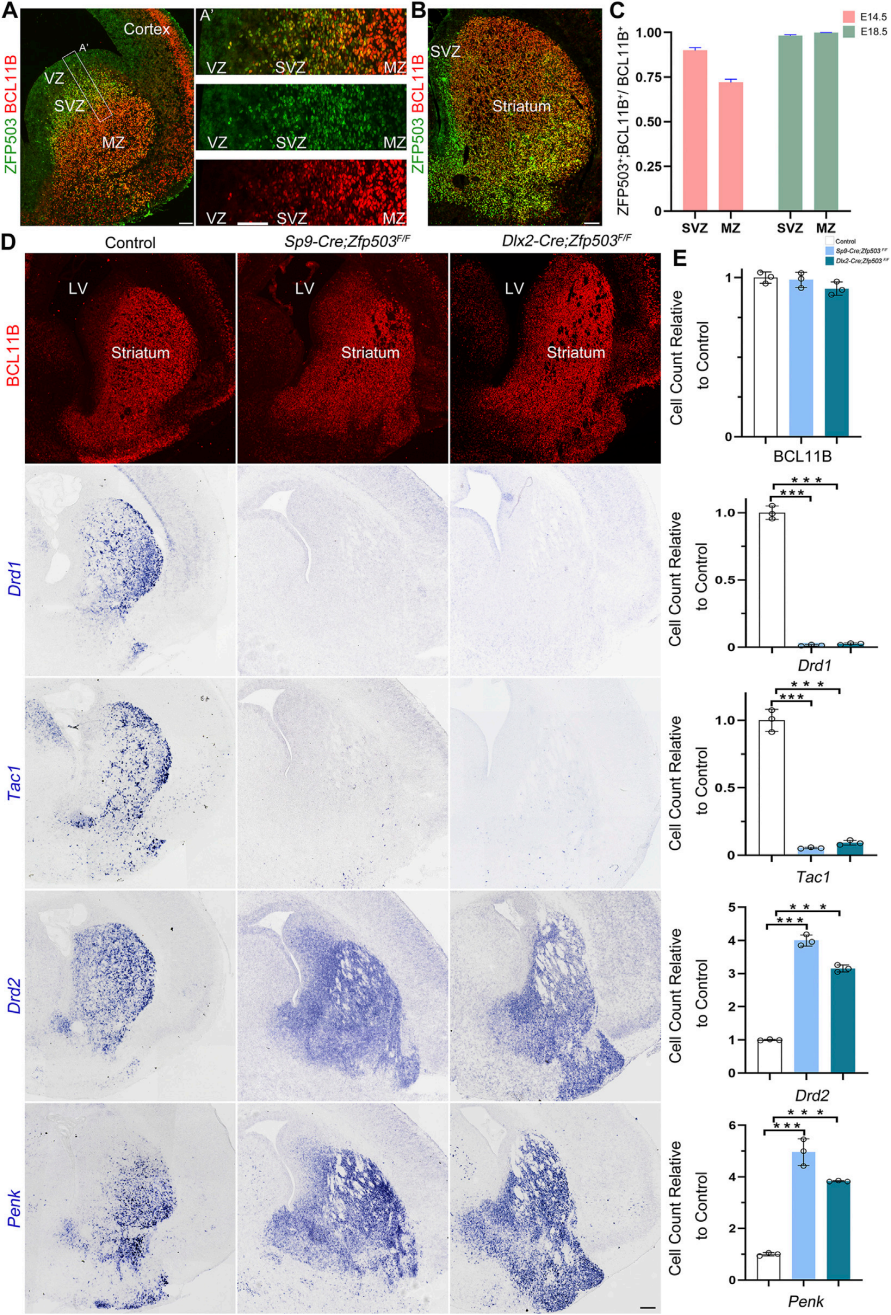
Fate switching of D1 MSNs into D2 MSNs in *Zfp503*-CKO mice. **(A)** ZFP503/BCL11B double-immunostained coronal hemisection at E14.5. **(A')** High magnification images of the boxed region in **(A)**. **(B)** ZFP503/BCL11B double-immunostained coronal hemisection at E18.5. **(C)** Quantification showing that most of the ZFP503-positive cells in the LGE were colabelled with BCL11B at E14.5 and E18.5. **(D)** BCL11B-positive cells, which represent the vast majority of striatal MSNs, were similar between *Zfp503*-CKO mice and control mice. *Drd1* and *Tac1* expression was significantly decreased whereas *Drd2* and *Penk* expression was markedly increased in the LGE at E18.5 in the *Zfp503*-CKO mice compared to the controls. **(E)** The quantification data of the **(D)**. (one-way ANOVA followed by Tukey–Kramer post-hoc test, ***P < 0.001, *n* = 3, mean ± SEM). Abbreviations: lateral ventricle (LV), mantle zone (MZ), ventricular zone (VZ), subventricular zone (SVZ). Scale bars: 100 µm in A; 100 µm in A’; 200 µm in **(D)**.

Previously, we and others reported that *Zfp503* is highly expressed in the LGE (Chang et al., 2004; Ko et al., 2013; Su et al., 2022), but the temporal expression of *Zfp503* during striatal development is not fully understood. Firstly, we performed double immunostaining of ZFP503 with the pan-striatal MSN marker BCL11B at E14.5 and E18.5 (Figures 1A,B). Our results showed that approximately 90% of BCL11B positive cells are ZFP503/BCL11B double-positive cells in the subventricular zone (SVZ), but the percentage was decreased to 70% in the mantle zone (MZ) at E14.5 (Figure 1C). Interestingly, nearly all BCL11B-positive cells were co-labelled with ZFP503 in the SVZ/MZ at E18.5 (Figures 1B,C). Next, we performed immunostaining in the developing mouse brain with the ZFP503 antibody. The expression of ZFP503 was detected in the LGE (striatal primordia) at E12.5 and E14.5 (Supplementary Figures S1A,B). A similar expression of ZFP503 was also observed in the LGE at E16.5 (Supplementary Figure S1C). At perinatal period, ZFP503 was present in the entire striatum, including the caudoputamen (CP), nucleus accumbens (NAc) and olfactory tubercle (OT) (Supplementary Figures S1D,E). These results indicated that *Zfp503* is highly expressed in the LGE during striatal MSN development.

To examine the role of *Zfp503* in regulating the fate of MSNs, we used two Cre lines (*Sp9-Cre* and *Dlx2-Cre*) to delete the expression of *Zfp503* in the basal ganglia. The phenotype of the *Zfp503* mutant mice was analyzed at E18.5. We found that there was no significant difference in BCL11B expression between control mice and *Zfp503*-CKO mice (Figures 1D,E). These results were further confirmed by our RNA-seq data (Supplementary Table 1) and were consistent with previous research (Chen et al., 2020). *Drd1* and *Tac1*, which are specifically expressed in D1 MSNs were dramatically decreased in the striatum of *Zfp503*-CKO mice compared to controls. In contrast, *Drd2* and *Penk*, which are specifically expressed in D2 MSNs, were markedly increased in *Zfp503*-CKO mice (Figures 1D,E). Next, we analysed the phenotype of the *Sp9-Cre; Zfp503* ^*F/F*^ (*Zfp503*-SCKO) mice at the transcriptional level. RNA-seq of the entire LGE was performed at E18.5. Again, our results showed that D1 MSN marker genes (*Drd1*, *Tac1*, *Isl1*, *Sox8,* and *Pdyn*) were significantly decreased. In contrast, D2 MSN marker genes (*Drd2*, *Penk*, *Six3*, *Grik3,* and *Gucy1a3*) were significantly increased (Supplementary Table 1). Taken together, our results indicated that striatal D1 MSNs are converted into D2 MSNs in the absence of *Zfp503*.

### Maturation of D1 MSNs seems faster than that of D2 MSNs

**FIGURE 2:**
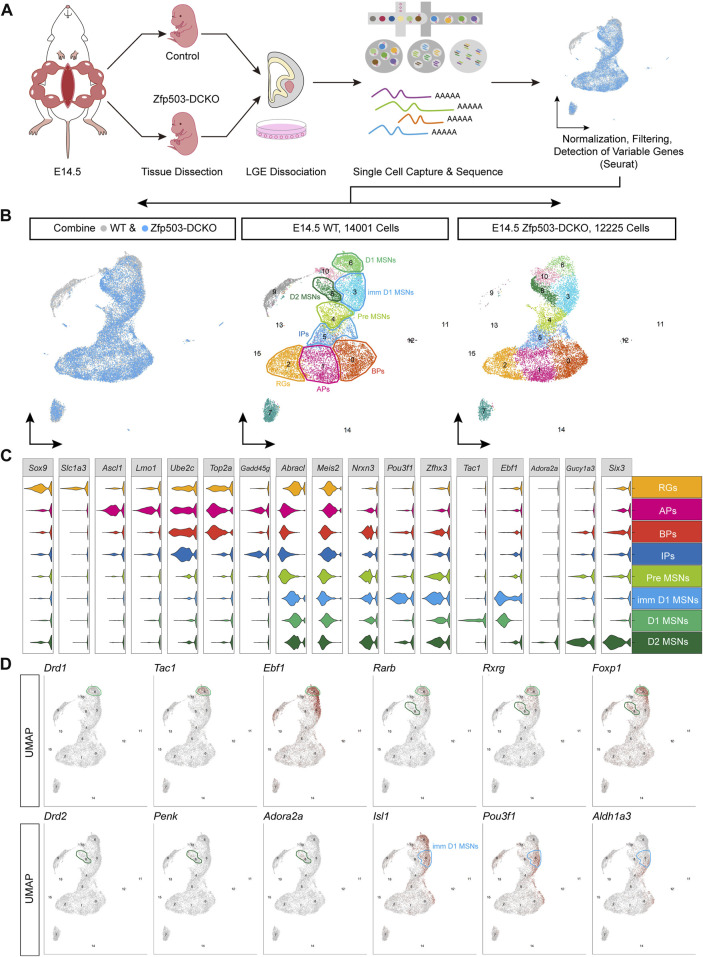
The maturation of D1 MSNs occurs earlier than that of D2 MSNs. **(A)** Workflow of scRNA-seq of LEG samples at E14.5. LGE tissues were dissected from embryos and dissociated into single-cell suspensions. Single cells were captured into droplets with the 10x platform. After sequencing, cells were classified by their transcriptomes. **(B)** UMAP showing 16 clusters (C0–C15); the major cell types are annotated in both the WT sample and the *Dlx2-Cre; Zfp503* ^*F/F*^ (*Zfp503*-DCKO) sample. **(C)** Violin plot showing cluster annotations, cell type assignments (y-axis) and the expression of 17 marker genes (x-axis). **(D)** Feature plots of the 12 marker genes in 16 different cell types in the WT sample. Abbreviations: Radial glia cells (RGs); apical progenitors (APs); basal progenitors (BPs); intermediate progenitors (IPs); premature MSNs (Pre-MSNs); immature D1 MSNs (Imm-D1 MSNs).

To investigate the mechanism by which *Zfp503* control fate switching between D1 and D2 MSNs, scRNA-seq experiments were performed to analyze LGE progenitors undergoing fate switching (Figure 2A). LGE samples from E14.5 *Zfp503*-DCKO (*Dlx2-Cre; Zfp503* ^*F/F*^, *n* = 3) mice were dissected, dissociated into single-cell suspensions, and sequenced using the 10X genomics platform. We also used the published scRNA-seq data (Li et al., 2022) of the wild type (WT) LGE at E14.5 from our lab. After removing outlier cells that had a high percentage of ribosomal or mitochondrial genes, 14,001 cells in the WT samples and 12,225 cells in the *Zfp503*-DCKO samples were used for analysis, with an average of 2,107 genes detected per cell (Figure 2B). Our initial clustering analysis revealed 16 (C0-C15) major cell populations with distinct gene expression patterns (Figure 2B; Supplementary Table 2). We used marker genes to define the identities of the 16 clusters more precisely, which resulted in eight discrete populations: RGs, APs, BPs, IPs, Pre-MSNs, imm-D1 MSNs, D1 MSNs, and D2 MSNs (Figure 2C; Supplementary Table 2). According to analysis of marker genes, we determined the identity of the remaining cell clusters: C7: striatal interneurons (*Lhx6*, *Lhx8*, *Sst*, and *Npy*); C9: cortical projection neurons (*Neurod6*, *Tbr1* and *Cux2*); C10: intercalated cells (*Zic1* and *Resp18*); C11: ependymal cells (*S100a11*, *Anxa2* and *Cryab*); C12: endothelial cells (*Cdh5* and *Cd34*); C13: blood cells (*Alas2*); C14: microglial cells (*Bcl2a1b*, *P2ry12* and *Trem2*); and C15: oligodendrocyte cells (*Olig1/2* and *Sox10*).

Next, we systematically characterized the gene features of the D1 and D2 MSNs. To date, the generation sequence of D1 and D2 MSNs during striatal development is not fully understood. Interestingly, we found very low expression levels of *Drd1* and *Drd2* suggesting that the D1 and D2 MSNs were not fully matured at E14.5 (Figure 2D). However, D1 MSN marker genes (*Tac1* and *Ebf1*) were highly expressed. In contrast, D2 MSN marker genes (*Penk* and *Adora2a*) were weakly expressed (Figure 2D). Furthermore, the pan-striatal MSN markers (*Rarb*, *Rxrg* and *Foxp1*) were highly expressed in D1 MSNs (C6) but not in D2 MSNs (C8) (Figure 2D). These results indicated that D1 MSN maturation seems faster than D2 MSN maturation. Recent studies indicated that *Isl1*, *Zfhx3* and *Zfp503* are expressed in the immature D1 MSNs (Ko et al., 2013; Zhang et al., 2019; Song et al., 2021; Ehrman et al., 2022; Li et al., 2022). Interestingly, we found that *Pou3f1* and *Aldh1a3* are specifically expressed in immature D1 MSNs (C3). Recently studies (Bocchi et al., 2022; Su-Feher et al., 2022) reported that *Pou3f1* and *Aldh1a3* are enriched in immature D1 MSNs. Thus, our findings and previous studies suggested that *Pou3f1* and *Aldh1a3* represent new markers for immature D1 MSNs.

### Blocking differentiation of LGE progenitors in *Zfp503*-DCKO mice

**FIGURE 3:**
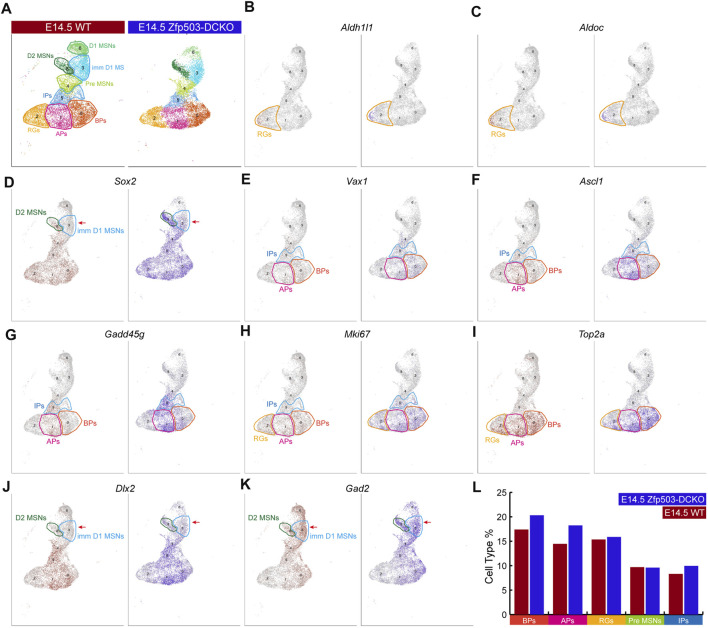
*Zfp503* regulates the differentiation of progenitor cells. **(A)** The major cell populations of the LGE at E14.5. **(B–C)** There were no significant differences in the expression of *Aldh1l1* and *Aldoc* between WT and *Zfp503*-DCKO mice. **(D–K)** The expression of *Sox2*, *Vax1*, *Ascl1*, *Gadd45g*, *Mki67*, *Top2a*, *Dlx2,* and *Gad2* was increased in the *Zfp503-*mutant sample compared to the WT sample. **(L)** The percentages of BPs, APs, and IPs were increased but the percentages of RGs and Pre-MSNs were not altered in the *Zfp503*-DCKO sample compared to the WT sample. Abbreviations: radial glia cells (RGs); apical progenitors (Aps); basal progenitors (BPs); intermediate progenitors (IPs); premature MSNs (Pre-MSNs); immature D1 MSNs (Imm D1 MSNs).

Next, we explored the mechanism underlying fate switching between D1 MSNs and D2 MSNs in *Zfp503*-DCKO (*Dlx2-Cre; Zfp503* ^*F/F*^) mice. Thus, we focused our further analysis on the major cell types in the LGE at E14.5 (Figure 3A). The radial glia cells (RGs) that expressed *Aldh1l1* and *Aldoc* were not significantly altered between *Zfp503*-DCKO mice and WT mice (Figures 3B,C,L). *Sox2* is highly expressed in the progenitors of the LGE and downregulated in immature neurons. Surprisingly, our results showed that *Sox2* expression was upregulated in most cell types, especially in immature MSNs in *Zfp503*-DCKO mice (Figures 3D,L). Recent studies reported that *Ascl1* and *Vax1* are highly expressed in basal progenitors (BPs) (Li et al., 2021; Wen et al., 2021; Bocchi et al., 2022), and a study showed that *Gadd45g* is a direct downstream target of *Ascl1* (Huang et al., 2010). These genes were increased in the *Zfp503*-DCKO mice (Figures 3E–G,L). We also found that the proliferating cells expressing *Ki67* (*Mki67*) and *Top2a* were increased in the *Zfp503*-DCKO mice compared to the WT mice (Figures 3H,I,L). Finally, our results showed that *Dlx2* and its direct downstream target *Gad2* were increased in MSNs (Figures 3J–L). In addition, we performed immunostaining of the progenitor cell markers, including PCNA, ASCL1 and KI67 at E14.5 to further support our findings. Our results showed that the number of the PCNA-, ASCL1-, and KI67-positive cells are increased in the *Zfp503*-DCKO mice compared to control mice (Supplementary Figures S2A–I). Taken together, these findings indicated that the differentiation of progenitors is blocked in *Zfp503*-DCKO mice.

### *Zfp503* promotes the D1 MSN identity and represses the D2 MSN identity

**FIGURE 4:**
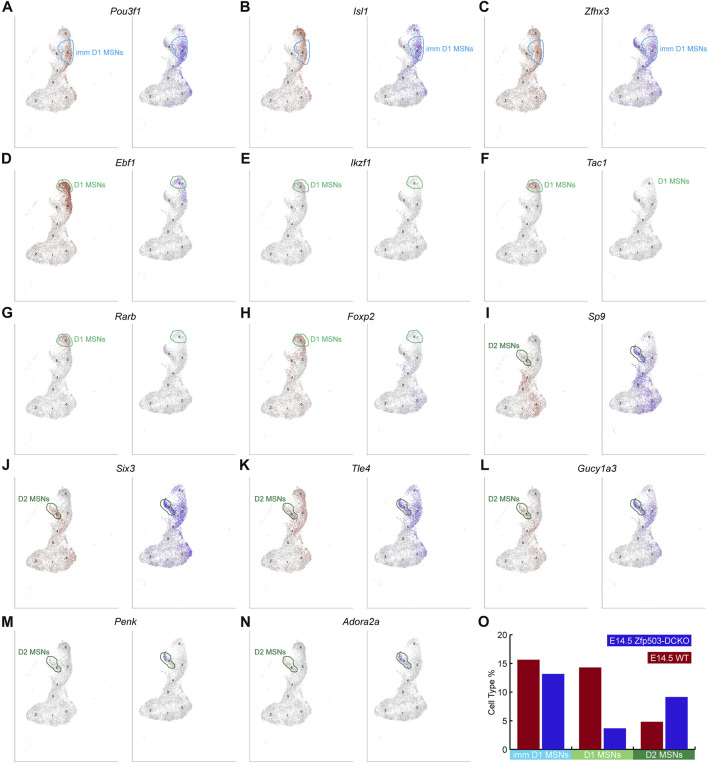
scRNA-seq reveals that *Zfp503* promotes the D1 MSN identity and represses the D2 MSN identity. **(A-C)** There were no significant changes in the expression of immature D1 MSN markers, including *Pou3f1*, *Isl1,* and *Zfhx3,* between *Zfp503*-DCKO mice and WT mice. **(D-H)** The expression of *Ebf1*, *Ikzf1*, *Tac1*, *Rarb,* and *Foxp2* was decreased in *Zfp503*-DCKO mice. **(I-N)** The expression of *Sp9*, *Six3*, *Tle4*, *Gucy1a3*, *Penk,* and *Adora2a* was increased in *Zfp503*-DCKO mice compared to WT mice. **(O)** The cell types percentage in *Zfp503*-DCKO mice and WT mice.

To further confirm our results regarding D1 and D2 MSN identity at the cell population level, we begin to analyze D1 and D2 MSN clusters in our scRNA-seq data. Previous work and our studies showed that *Pou3f1*, *Isl1* and *Zfhx3* are expressed in immature D1 MSNs (Ehrman et al., 2013; Lu et al., 2014; Zhang et al., 2019). We found that the expression of these genes in the immature D1 MSN cluster was not significantly changed between WT mice and *Zfp503*-DCKO mice (Figures 4A–C,O). However, *Ebf1*, *Ikzf1*, *Tac1*, *Rarb,* and *Foxp2*, which were highly expressed in D1 MSNs were reduced in the D1 MSN cluster of *Zfp503*-DCKO mice compared to WT mice (Figures 4D–H,O). Our previous studies showed that the TFs *Sp9* and *Six3* are required for the differentiation of D2 MSNs (Zhang et al., 2016; Xu et al., 2018; Song et al., 2021). In the absence of *Zfp503*, these genes were upregulated in D2 MSNs (Figures 4I,J). Furthermore, *Tle4* and *Gucy1a3* tend to be expressed in D2 MSNs, and our results showed that these genes were upregulated in D2 MSNs and immature D1 MSNs in *Zfp503*-DCKO mice (Figures 4K–L). In addition, the specific D2 MSN markers *Penk* and *Adora2a* were upregulated in *Zfp503*-DCKO mice compared to WT mice (Figures 4M–O). Again, our findings indicated that D1 MSNs undergo fate switching into D2 MSNs at the cell population level in *Zfp503*-DCKO mice.

### *Zfp503* represses the D2 MSN identity by regulating *Sp8/9* expression

**FIGURE 5:**
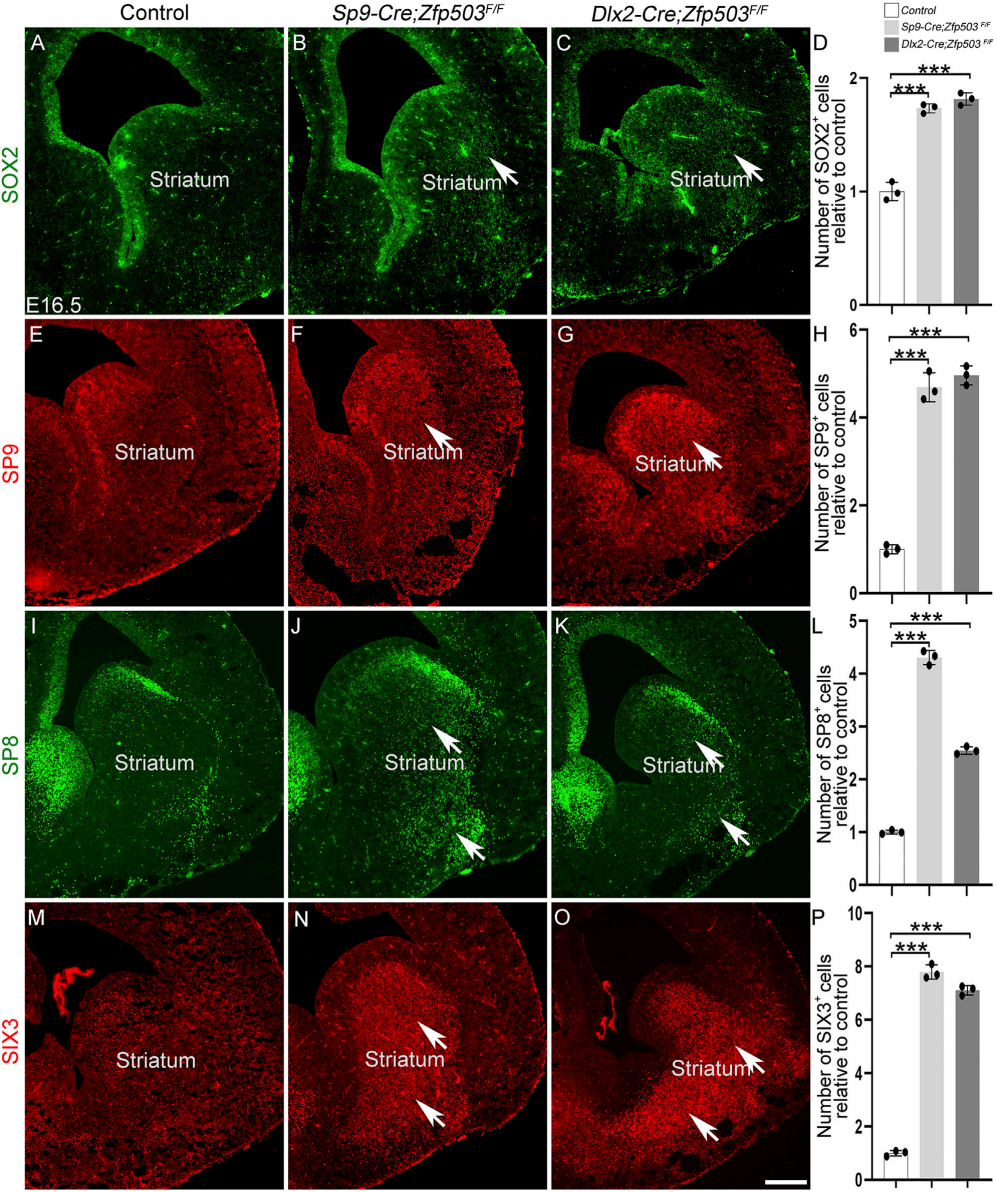
Abnormal expression of precursor marker genes in *Zfp503*-CKO mice at E16.5. **(A–D)** The expression of *SOX2* was increased in the entire striatum of *Zfp503*-CKO mice at E16.5. **(E–L)** The transcription factors *Sp8* and *Sp9* were increased in the striatum of *Zfp503*-CKO mice at E16.5. **(M–P)** *Six3* expression was present in the whole striatum in *Zfp503*-CKO mice, but not in control mice. One-way ANOVA followed by the Tukey–Kramer post hoc test, ***P < 0.001, *n* = 3, mean ± SEM. Scale bar: 200 µm in O for **(A–O)**.

**FIGURE 6:**
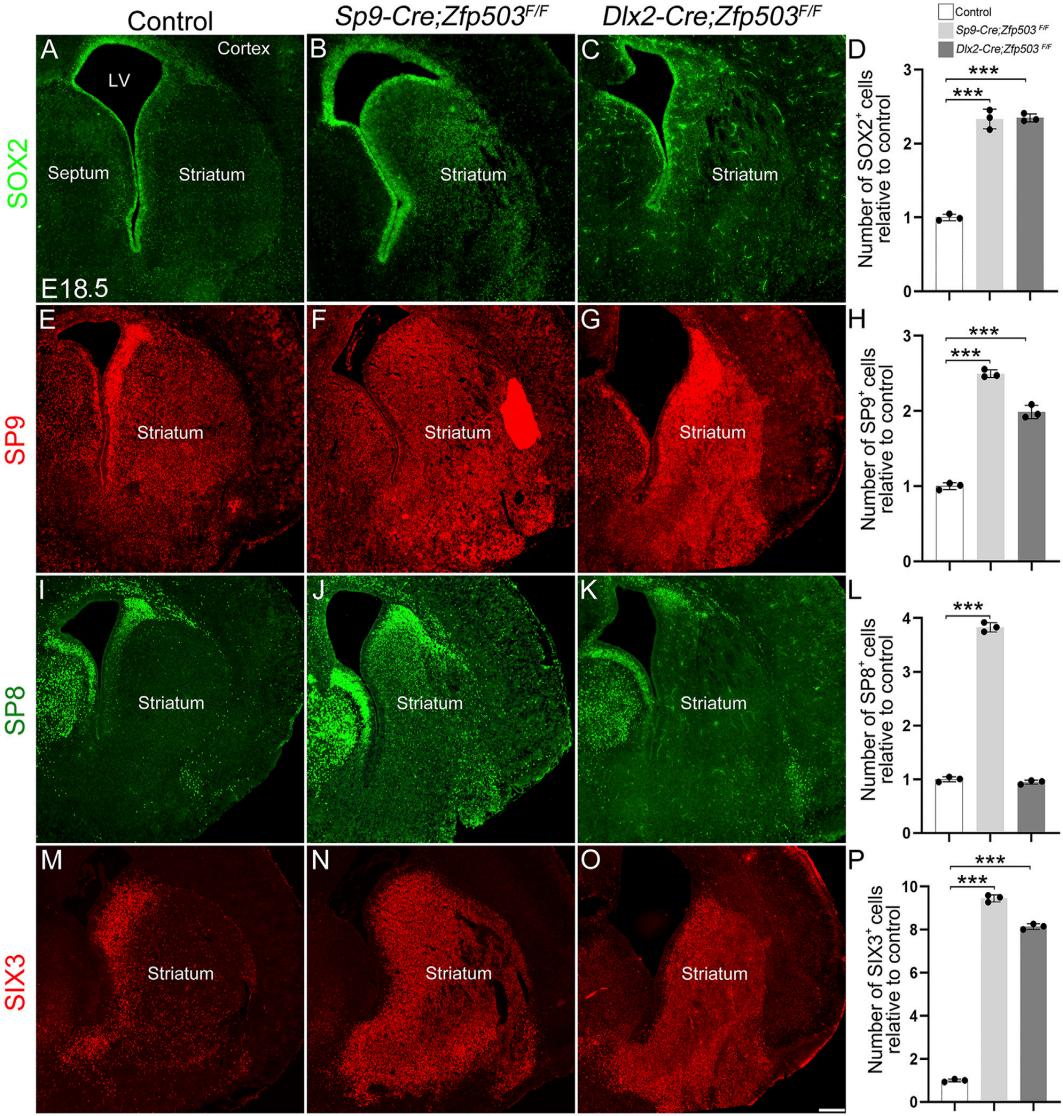
Abnormal expression of precursor marker genes in the striatum of *Zfp503*-CKO mice at E18.5. **(A–D)** The expression of *SOX2* was increased in the entire striatum of *Zfp503* mutants at E18.5. **(E–L)** The transcription factors *Sp8* and *Sp9* were increased in the striatum of *Zfp503*-CKO mice at E18.5. **(M–P)** *Six3* expression was present in the whole striatum in *Zfp503*-CKO mice, but not in control mice. Abbreviations: lateral ventricle (LV). One-way ANOVA followed by Tukey–Kramer post hoc test, ***P < 0.001, *n* = 3, mean ± SEM. Scale bar: 200 µm in O for **(A–O)**.

To gain insights into the mechanism by which *Zfp503* regulates the fate switching of striatal MSNs, we performed immunostaining at E16.5 and E18.5. Previous studies reported that TF *Sox2* was mainly expressed in the neural precursor cells and regulated multiple aspects of telencephalic development (Cimadamore et al., 2013; Feng et al., 2013; Feng and Wen, 2015). Our previous studies demonstrated that the TFs *Sp8/9* and *Six3* are required for the differentiation of D2 MSNs (Zhang et al., 2016; Xu et al., 2018; Song et al., 2021). Here, we found that *SOX2* expression was significantly upregulated in the whole striatum at E16.5 and E18.5 in *Zfp503*-CKO mice compared to controls (Figures 5A–D, 6A–D). Considering that striatal MSNs express relatively mature markers, such as *Drd2* and *Penk* (Figure 1D), we speculate that the striatal MSNs is not well differentiated due to blockade of differentiation at the progenitor stage. Moreover, our results showed that the TFs *Sp8/9* were dramatically increased in the striatum at E16.5 and E18.5 in the *Zfp503*-CKO mice compared to controls (Figures 5E–L, 6E–L). Interestingly, *Sp8* expression was significantly increased at E16.5 (Figures 5I–L) and E14.5 (data not shown) in both Cre lines, but the expression of *Sp8* was not altered at E18.5 in the *Dlx2-Cre* line (Figures 6I–L). Due to the *Dlx2-Cre* deleted *Zfp503* expression mainly in LGE radial glial cells and the *Sp9-Cre* deleted *Zfp503* expression mainly in progenitors which were located in the LGE SVZ. Therefore, we speculate that *Zfp503* may exert its function in radial glial cells. It may be that *Zfp503* promotes cell cycle exit of radial glial cells, which leads to *Sp8* expression showing the different phenotypes at different time points. In addition, *Six3*, a direct downstream target of *Sp8/9* was significantly increased in *Zfp503*-CKO mice at E16.5 and E18.5 (Figures 5M–P, 6M–P). Taken together, these findings suggest that *Zfp503* represses the D2 MSN identity by repressing the expression of *Sp8/9*.

### Overexpression of *Zfp503* promotes the D1 MSN identity and suppress the D2 MSN identity

**FIGURE 7:**
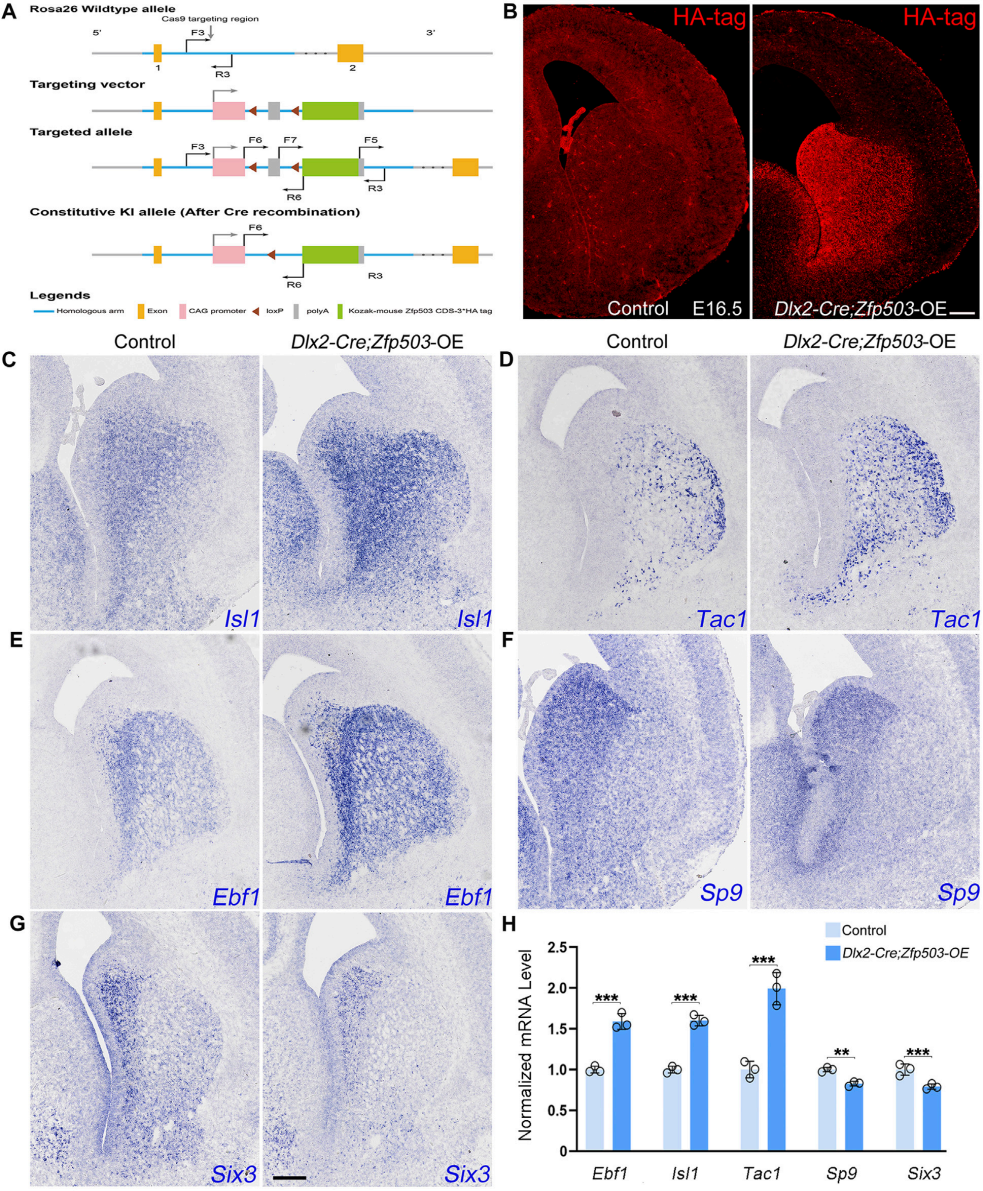
The D1 MSN identity is enhanced and the D2 MSN identity is compromised in *Zfp503*-overexpressing mice. **(A)** Generation of the *Zfp503* overexpression allele. A CAG promoter followed by a *Zfp503* CDS and three HA‐tag sequences was inserted into the *Rosa-26* site with the CRISPR/Cas9 strategy. **(B)** HA-tag was expressed in the septum, striatum and cortical interneurons, which is consistent with the *Dlx2* expression in the forebrains. **(C–E)** The expression of *Isl1*, *Tac1,* and *Ebf1,* which are specifically expressed in D1 MSNs, was increased in *Dlx2-Cre; Zfp503-OE* mice at E16.5 compared to controls. **(F–G)** The expression of the TFs *Sp9* and *Six3* was reduced in *Dlx2-Cre; Zfp503-OE* mice compared to controls. **(H)** Histogram shows quantification data of the above markers in the *Dlx2-Cre; Zfp503-OE,* and control mice at E16.5 (unpaired two-tailed Student’s t-test, **P < 0.01, ***P < 0.001, *n* = 3 mice per group, mean ± SEM). Scale bar: 100 µm in B for **(B)**, 100 µm in G for (C‐G).

In addition to investigating the effects of *Zfp503* loss-of-function, we also explored the effects of *Zfp503* gain-of-function in striatal MSNs. First, we generated a *Zfp503* overexpression allele (*Zfp503*-OE). A CAG promoter followed by a *Zfp503* CDS and three HA-tag sequences was inserted into the *Rosa-26* site with the CRISPR/Cas9 strategy (Figure 7A). Then, we crossed *Zfp503*-OE mice with *Dlx2-Cre* mice to obtain *Zfp503* overexpressing mice (*Dlx2-Cre; Zfp503-*OE). *Dlx2-Cre; Zfp503-OE* embryos showed robust Ha-tag signals in *Dlx2*-expressing regions along the dorso-ventral (DV) axis, including the septum, striatum, and cortex interneuron (Figure 7B). To study the function of *Zfp503* in regulating the cell identity of striatal MSNs, we used *in situ* hybridization to detect the expression of D1 MSN and D2 MSN markers. Consistent with our expectations, the expression of *Isl1*, *Tac1,* and *Ebf1* was increased in the *Zfp503* overexpressing mice **(** Figures 7C–E,H **)**. In contrast, TFs *Sp9* and *Six3*, which control the differentiation of D2 MSNs were reduced in *Zfp503* overexpressing mice compared to control mice (Figures 7F–H). In addition, the RNA-seq data showed that the D1 MSN identity was enhanced and the D2 MSN identity was compromised in the *Dlx5/6-Cre*; *Zfp503*-OE mice compared to the control mice (Supplementary Table 3). Overall, our findings suggested that *Zfp503* promotes the D1 MSN identity and represses the D2 MSN identity.

## Discussion

The generation of striatal D1 versus D2 MSNs is controlled by different TFs. In this study, we found that the TF *Zfp503* is intensely expressed in MSNs during striatal development. The absence of *Zfp503* resulted in fate switching of D1 MSNs into D2 MSNs. Single-cell RNA-seq and histochemical analyses revealed that *Zfp503* promotes the D1 MSN identity and represses the D2 MSN identity. Mechanistically, our results suggest the upregulation of the TFs *Sp8/9* is the key factor leading to the switching of D1 MSNs to the D2 MSNs in *Zfp503*-CKO mice. Overall, we speculate that TF *Zfp503* controls the fate of D1 MSNs by repressing *Sp8/9* expression during striatal development.

### D1 MSNs and D2 MSNs are generated in distinct domains in the LGE

Understanding the mechanisms underlying neuronal fate acquisition is a major challenge in developmental neurobiology, because there are many distinct regions and different progenitors in the mammalian telencephalon, particularly in the subpallium (Yun et al., 2001; Corbin et al., 2003; Stenman et al., 2003; Flames et al., 2007; Wen et al., 2021). More recent evidence has shown that TF *Zfp503* expression is enriched in the ventral parts of the vLGE (Chen et al., 2020), while the TF *Six3* expression preferentially present in the dorsal parts of the vLGE at an early stage (Xu et al., 2018). Furthermore, the differentiation of D1 MSNs and D2 MSNs is completely blocked in *Zfp503*-CKO and *Six3*-CKO mice, respectively (Xu et al., 2018; Soleilhavoup et al., 2020). These results support that D1 MSNs are generated from the ventral part of the vLGE, in contrast to D2 MSNs which are derived from the dorsal part of the vLGE.

Retinoic acid (RA) has been implicated as an extrinsic signal regulating the differentiation of distinct cell types, including specific neuronal subtypes (Toresson et al., 1999; Chatzi et al., 2011; Cunningham and Duester, 2015; Rataj-Baniowska et al., 2015; Podleśny-Drabiniok et al., 2017). RA is derived from vitamin A through a two-step enzymatic process, employing retinol dehydrogenase (*Rdh10*) for the oxidation of retinol to retinaldehyde, and the retinaldehyde dehydrogenases *Aldh1a1*, *Aldh1a2*, and *Aldh1a3* for the oxidation of retinaldehyde to RA, which then functions as a ligand for nuclear RA receptors (Chatzi et al., 2011). During striatal development, *Aldh1a3* is predominantly expressed in LGE (striatal primordium) progenitors and is nearly absent in adjacent structures of the medial ganglionic eminence and cerebral cortex (Toresson et al., 1999; Molotkova et al., 2007). Its expression is regulated by *Gsx2* and *Meis2* (Waclaw et al., 2004; Su et al., 2022). In fact, the expression of the *Aldh1a3* is located in the ventral vLGE (Li et al., 2000; Smith et al., 2001; Waclaw et al., 2004; Chatzi et al., 2011). The roles of RA in the LGE have been investigated, including promoting striatal neurogenesis and neuronal differentiation (Molotkova et al., 2007; Liao et al., 2008; Urban et al., 2010; Al Tanoury et al., 2014; Rataj-Baniowska et al., 2015). The absence of *Aldh1a3* leads to abnormal differentiation of striatal MSNs (Chatzi et al., 2011). Interestingly, RA can promote the expression of *Zfp503* in the LGE at the early stage of striatal development (Urban et al., 2010). These studies support that RA can induce *Zfp503* expression in ventral vLGE progenitors at an early stage. Therefore, we speculate that the progenitors located in the ventral vLGE tend to give rise to D1 MSNs. In contrast, the progenitors located in the dorsal vLGE tend to generate D2 MSNs.

### *Foxp2* expression is inhibited by *Six3* in the D2 MSN lineage

The Forkhead box P2 (FOXP2) has been identified as a gene related to neurodevelopmental disorders, and it was also the first gene to be associated with vocal functions in songbirds and rodents (Bacon and Rappold, 2012; Chiu et al., 2014). Indeed, mutations in the FOXP2 gene have been identified in patients with a severe speech and language disorders (Lai et al., 2001). Naturally, *Foxp2* is enriched in the striatum and controls the differentiation of striatal MSNs (Lai et al., 2001; Chiu et al., 2014; Chen et al., 2016; Fong et al., 2018; van Rhijn et al., 2018). The mechanisms, however, of regulating *Foxp2* expression in the striatum are largely unknown. More recent evidence supports that *Foxp2* is highly expressed in D1 MSNs and weakly expressed in the D2 MSNs (Liu et al., 2018; Tinterri et al., 2018; van Rhijn et al., 2018; Wei et al., 2019). In this study, we found that the expression of *Foxp2* was significantly reduced in *Zfp503*-DCKO mice (Figure 4H; Supplementary Table 1). In contrast, the expression of *Six3* was present in the whole striatum (Figures 6M–O). *Six3* is known as a key factor for the generation of D2 MSNs (Xu et al., 2018; Song et al., 2021). Thus, we speculated that *Six3* represses the expression of *Foxp2* in D2 MSNs. Actually, in our recent works and unpublished data, we found that *Foxp2* expression is upregulated in *Six3* mutant mice and downregulated in *Six3* overexpressing mice (Xu et al., 2018; Song et al., 2021). Therefore, our findings suggested that *Foxp2* expression is closely regulated by *Six3* in D2 MSNs.

Overall, our findings have demonstrated that *Zfp503* is critical for the fate acquisition of D1 versus D2 MSNs during striatal development. *Zfp503* appears to repress the expression of the TF *Sp8/9* in precursor cells to control the fate of D1 MSNs. The results of this work will deepen our understanding of fate determination in striatal MSNs and open up new opportunities for translational investigations of degenerative diseases, such as PD and HD.

## Materials and methods

### Animals

All experiments conducted in this study were in accordance with guidelines from Fudan University, China. *Zfp503* ^*F/+*^ (Su et al., 2022), *Sp9-Cre* (Zhang et al., 2016), and *Dlx5/6-CIE* (Liu et al., 2018) mice have been previously described. *Dlx2-Cre* mice were generated *via* the CRISPR/Cas9 strategy. IRES elements and *Cre* sequences were knocked into the coding region of exon 3 of *Dlx2*. Genotyping of the *Dlx2-Cre* constitutive knock-in (KI) allele was performed by PCR using the following primers:

F1: 5′- CCT​CGG​CCT​TTC​TGG​GAA​ACT​AC-3′;

R1: 5′- CTT​GCA​GGT​ACA​GGA​GGT​AGT​CC-3′;

R2: 5′- TTG​GCA​CTA​AAG​GAT​CCC​ACG​AG-3′. These primers yielded bands of 390 and 501 bp for the WT and knock-in (KI) alleles, respectively. Rosa26-Zfp503-OE/+ mice that conditionally overexpressed Zfp503 were generated by CRISPR/Cas9 techniques (Figure 7A). An expression vector containing CAG-promoter-Flox-PolyA STOP-Flox-Kozak-mouse Zfp503 CDS-3 XHA tag-PolyA was knocked into the Rosa26 locus. With this strategy, the Zfp503-HA tag fusion protein was continuously expressed in CRE recombinase-positive cells. In cells after CRE recombination, both the HA tag and the Zfp503 protein were specifically detected. Genotyping of the Zfp503-OE allele was performed by PCR using the following primers:

WT-F3: 5′-CAC​TTG​CTC​TCC​CAA​AGT​CGC​TC-3′;

WT-R3: 5′-ATA​CTC​CGA​GGC​GGA​TCA​CAA-3′;

OE-F5: 5′-GCA​TCT​GAC​TTC​TGG​CTA​ATA​AAG-3′. These primers yield bands of 453 and 633 bp for the WT and overexpression alleles, respectively. All mice were maintained in a mixed genetic background of C57BL/6J and CD1. The day of vaginal plug detection was considered E0.5, and the day of birth was defined as P0. Both male and female mice were used in all experiments.

### Tissue preparation

Tissue preparation was performed as previously described (Yang et al., 2021).

### Immunohistochemistry

In this study, 6-μm and 12-μm thick frozen sections were used for immunostaining. The sections were washed with 0.05 M Tris buffered saline (TBS) for 10 min, (If necessary, the sections were immersed in antigenic repair solution(pH = 6.0) and boiled before the Triton step, and cooled naturally to room temperature (RT) incubated in Triton X-100 (0.5% in 0.05 M TBS) for 30 min at RT, and then incubated with blocking solution (10% donkey serum + 0.5%; Triton-X-100 in 0.05 M TBS, pH 7.2) for 2 h at RT. The primary antibodies were diluted in 10% donkey serum blocking solution, incubated overnight at 4°C and then rinsed three times with 0.05 M TBS. Secondary antibodies matching the appropriate species were added and incubated for 2-4 h at RT. Fluorescently stained sections were then washed three times with 0.05 M TBS. This was followed by 4′,6-diamidino-2-phenylindole (DAPI) (Sigma-Aldrich, 200 ng/ml) staining for 5 min, and the sections were then cover-slipped with Gel/Mount (Biomeda).

### *In situ* RNA hybridization

In situ hybridization (ISH) was performed on 20-μm cryosections using digoxigenin riboprobes as previously described (Su et al., 2022). Probes were made from P0 WT mouse brain cDNA amplified by PCR.

### RNA-seq

LGE samples of E16.5 *Zfp503*-OE, E18.5 *Zfp503*-CKO and littermate control mice were dissected (*n* > = 3 for each group). Total RNA was purified with a Mini RNA Isolation Kit (Zymo) followed by library generation according to the manufacturer’s protocol (Illumina TruSeq Stranded Total RNA Library Prep Kit with Ribo-Zero Mouse). Fragment size distribution was assessed using a Bioanalyzer 2100. The concentration of the libraries was measured using a Kapa Library Quantification Kit. The purified libraries were sequenced on a HiSeq 4000 platform. Gene expression levels were reported as fragments per kilobase of exon per million fragments mapped (FPKM) values. Genes with a *p* value < 0.05 were considered to be differentially expressed.

### Tissue processing for scRNA-seq

Embryonic mouse (E14.5) brains were quickly collected and placed in HBSS. The LGE samples were dissected and incubated in 1 mg/ml papain in HBSS for 20 min at 37°C. The tissues were gently dissociated into a single-cell suspension by pipetting. Cells were centrifuged and washed twice, filtered through Flowmi Tip 40 μM strainers, and resuspended in HBSS+ 0.04% BSA. Cell viability was assessed by trypan blue exclusion.

### sc-RNA-seq and analysis

The Chromium Droplet-based Sequencing Platform (10X Genomics) was used to generate scRNA-seq libraries, following the manufacturer’s instructions (manual document part number: CG00052 Rev C). The cDNA libraries were purified, quantified using an Agilent 2100 Bioanalyzer, and sequenced on an Illumina HiSeq4000. High quality sequences (clean reads) were obtained by removing low quality sequences and joints. The clean reads were then processed with Cell Ranger software to obtain quantitative information on gene expression. Genes expressed in < 3 cells and cells with < 750 detected genes were filtered out. Cells with >10% mitochondrial genes were also filtered out. The global-scaling normalization method “Log Normalize” was applied to the raw read counts generated by 10X Cell Ranger to normalize the gene expression measurements for each cell based on the total expression. The log-transformed normalized single-cell expression values were used for differential expression tests. Potential sources of variation, including technical noise, batch effects, and biological sources of variation such as cell-cycle stage, were removed to improve downstream dimensionality reduction and clustering. We regressed gene expression on the number of detected molecules per cell and the cell-cycle stage score. The scaled z-scored residuals were used for principal component analysis (PCA). Statistically significant principal components determined by a resampling test were kept for uniform manifold approximation and projection (UMAP) analysis. Differentially-expressed genes (DEGs) among clusters were identified by comparing cells in each cluster against all other cells with the likelihood-ratio test. Gene A was defined as a biomarker of cluster X if it was detected in ≥25% cells, and had an adjusted *p-value* < 5%, and fold change ≥2 between cells of cluster X and all other cells. All these analyses were performed in Seurat v3.2 (https://satijalab.org/seurat/).

### Image acquisition and statistical analysis

Images for quantitative analyses were acquired using an Olympus VS 120 microscope with a 20X objective. Bright-field images were acquired using an Olympus VS 120 microscope with a 10X objective. Images were merged, cropped and optimized in Adobe Photoshop CC without distorting the original information. Analyses were performed using GraphPad Prism 6.0, Microsoft Excel and the R language. Unpaired two-tailed *t-test* or one-way ANOVA followed by the Tukey–Kramer post-hoc test was used to determine statistical significance. All quantification results are presented as the mean ± SEM. Differences with *p-values* < 0.05 were considered significant.

For quantification of BCL11B^+^ and ZFP503^+^ cells in the striatum of Wild type at E14.5 and E18.5, four anatomically matched 6-μm thick coronal sections were selected (*n* = 3 mice per group). We counted BCL11B^+^ and ZFP503^+^ cells in the striatum under a 20X objective lens. The striatum was delineated by DAPI staining. The numbers of BCL11B^+^ and ZFP503^+^ cells per section for each LGE SVZ were presented as the relative to these in the control group.

For quantification of BCL11B^+^ cells in the striatum of control and two *Zfp503*-CKO mice at E18.5, four anatomically matched 12-μm thick coronal sections were selected (*n* = 3 mice per group). We counted BCL11B^+^ cells in the striatum under a 20X objective lens. The striatum was delineated by DAPI staining. The numbers of BCL11B^+^ cells per section for each striatum were presented as the relative to these in the control group.

For quantification of *Drd1* ^+^, *Tac1* ^*+*^ *, Penk* ^*+*^, and *Drd2* ^*+*^ cells in the striatum of control and two *Zfp503*-CKO mice at E18.5, four anatomically matched 20-μm thick coronal sections were selected (*n* = 3 mice per group). We counted *Drd1* ^*+*^ *, Tac1* ^*+*^ *, Penk* ^*+*^ *,* and *Drd2* ^*+*^ cells in the striatum under a 10X objective lens. The numbers of *Drd1* ^*+*^ *, Tac1* ^*+*^ *, Penk* ^*+*^ *,* and *Drd2* ^*+*^ cells per section for each striatum were presented as the relative to these in the control group.

For quantification of SIX3^+^, SOX2^+^, and SP9^+^ cells in the striatum of control and two *Zfp503*-CKO mice at E16.5 and E18.5, four anatomically matched 12-μm thick coronal sections were selected (*n* = 3 mice per group). We used the histogram tool in Adobe Photoshop CC to calculate the fluorescence intensity of SIX3^+^, SOX2^+^, and SP9^+^ cells in the striatum. Data were presented in proportion to the control group.

For quantification of SP8^+^ cells in the striatum of control and two *Zfp503*-CKO mice at E16.5 and E18.5, four anatomically matched 12-μm thick coronal sections were selected (*n* = 3 mice per group). We counted SP8^+^ cells in the striatum under a 20X objective lens. The striatum was delineated by DAPI staining. The numbers of SP8^+^ cells per section for each striatum were presented as the relative to these in the control group.

For quantification of *Ebf1* ^+^, *Isl1* ^+^, *Six3* ^+^, and *Sp9* ^+^ cells in the striatum of control and *Dlx2-Cre; Zfp503*-OE mice at E16.5, four anatomically matched 12-μm thick coronal sections were selected (*n* = 3 mice per group). We used the Adobe Photoshop CC to invert the image, and then used histogram tool in Adobe Photoshop CC to calculate the fluorescence intensity of *Ebf1* ^*+*^ *, Isl1* ^*+*^ *, Six3* ^*+*^ *,* and *Sp9* ^*+*^ cells in the striatum. Data were presented in proportion to the control group.

For quantification of *Tac1* ^*+*^ cells in the striatum of control and *Dlx2-Cre; Zfp503*-OE mice at E16.5, four anatomically matched 12-μm thick coronal sections were selected (*n* = 3 mice per group). We counted *Tac1* ^*+*^ cells in the striatum under a 10X objective lens. The numbers of *Tac1* ^*+*^ cells per section for each striatum were presented as the relative to these in the control group.

For quantification of PCNA^+^, KI67^+^, and ASCL1^+^ cells in the striatum of control and *Zfp503*-DCKO mice at E14.5, four anatomically matched 12-μm thick coronal sections were selected (*n* = 3 mice per group). We used the histogram tool in Adobe Photoshop CC to calculate the fluorescence intensity of PCNA^+^, KI67^+^, and ASCL1^+^ cells in the striatum. The striatum was delineated by DAPI staining. Data were presented in proportion to the control group.

## Data Availability

The datasets presented in this study can be found in online repositories. The names of the repository/repositories and accession number(s) can be found below: https://www.ncbi.nlm.nih.gov/geo/, GSE202439.
