# Supplementary material for: The transcription factor Zfp503 promotes the D1 MSN identity and represses the D2 MSN identity
Source: Front Cell Dev Biol. 2022 Aug 23;10:948331. doi: 10.3389/fcell.2022.948331 (PMC9445169; doi:10.3389/fcell.2022.948331)
Supplement: Supplementary file 4 [file Image1.pdf]

Supplementary

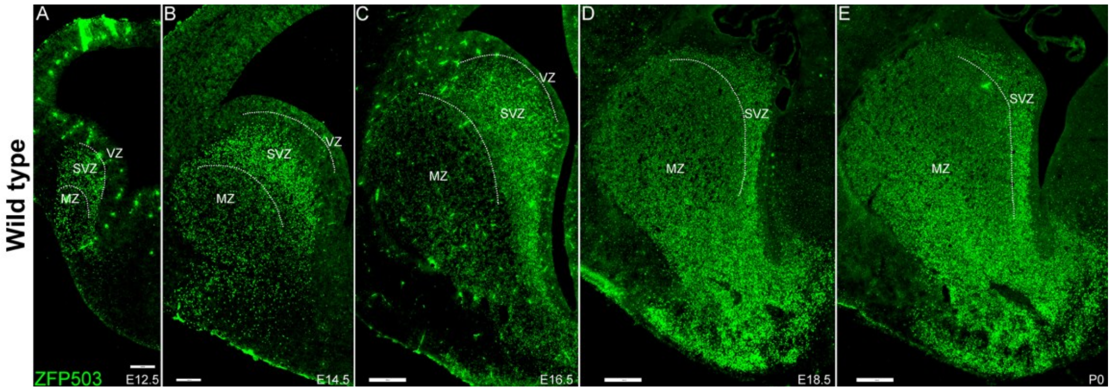

FIGURE S1. Ontogeny of Zfp503 protein expression in developing striatum.

(A-E) ZFP503 protein is selectively detected in the LGE (striatal primordia) at E12.5 (A), E14.5 (B), E16.5 (C), E18.5 (D) and P0 (E). Scale bar: 200 μm in A-E.

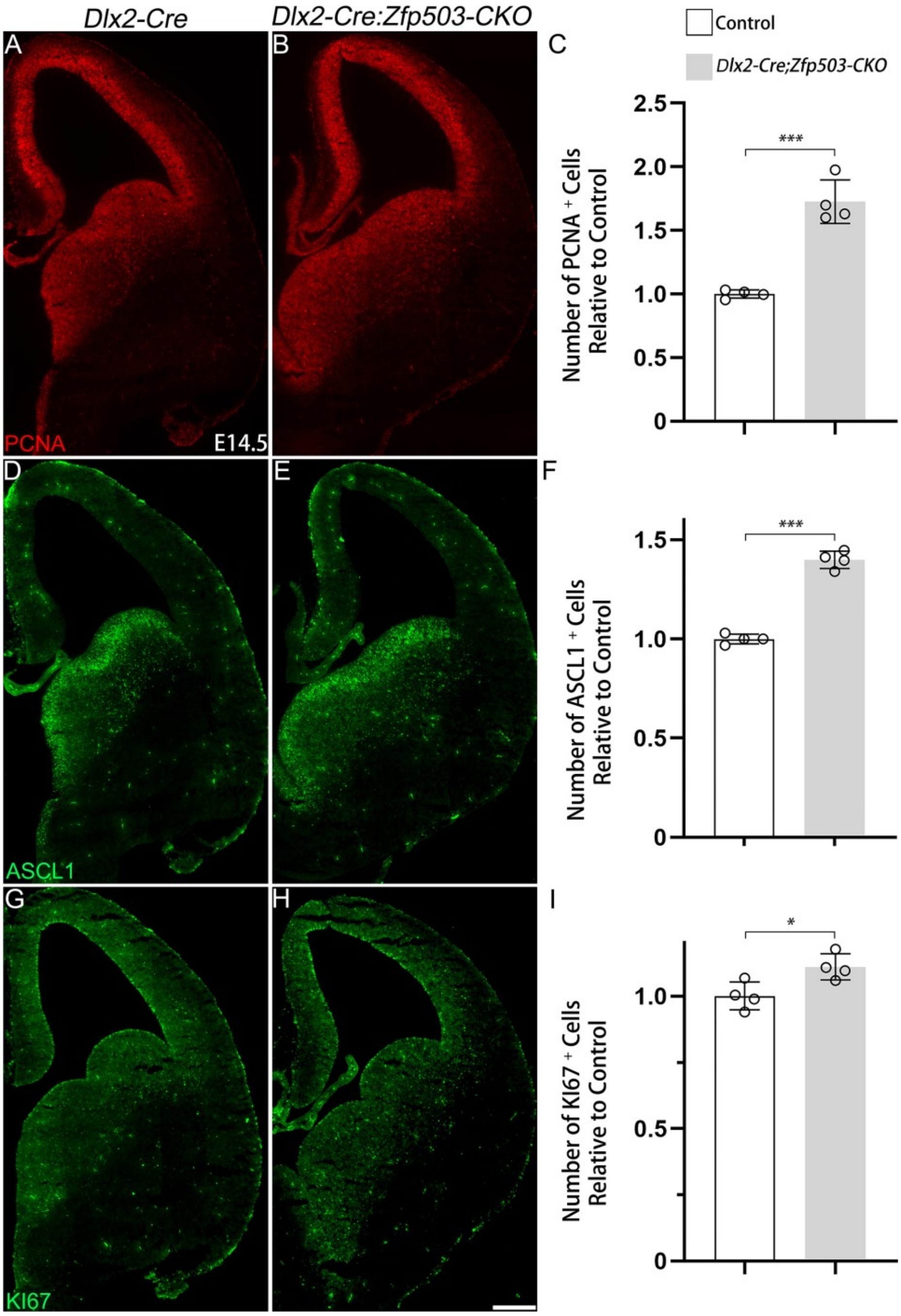

FIGURE S2. The differentiation of the progenitor cells was affected in the *Zfp503*-DCKO mice. (A-I) The number of the PCNA-, ASCL1- and KI67-positive cells was significantly reduced in the *Zfp503*-DCKO mice compared to the control mice. (unpaired two-tailed Student's t-test, *P < 0.05, ***P < 0.001, n = 4 mice per group, mean ± SEM). Scale bar: 500 μm in H for A-H.
